# A Multi-Dimensional Examination of Foraging Habitat Use by Gray Whales Using Long Time-Series and Acoustics Data

**DOI:** 10.3390/ani12202735

**Published:** 2022-10-12

**Authors:** Rianna E. Burnham, David A. Duffus

**Affiliations:** Whale Research Lab, Department of Geography, University of Victoria, Victoria, BC V8W 2Y2, Canada

**Keywords:** foraging intensity, passive acoustic monitoring, predator-prey, predictive habitat use, site fidelity, top-down forces

## Abstract

**Simple Summary:**

Long term data on the number and location of foraging gray whales on the west coast of Vancouver was used to understand the rate of return and residency time of whales to certain areas. From this data, areas of increased use by foraging whales were determined, and patterns in the way the areas were used by the whales were seen. Whale location data showed them using prey patches and moving to other patches to allow the first to recover, before then returning to feed on them again later in the summer. Calves that follow their mother in their first migration were likely to return to the same site in the years after weaning. This suggests a maternal aspect to the use of foraging areas. Little is known about how whales detect prey; the use of acoustics was considered here, with call types differing between times when prey was more abundant and whales were feeding in close proximity, to those when foraging whales were more distant to each other. This suggests there may be a social aspect to the calling by gray whales in foraging areas.

**Abstract:**

Gray whales (*Eschrichtius robustus*) show high site fidelity to feeding and breeding areas. The whales’ annual cycle could be learned or be driven by factors such as prey abundance or ocean conditions. Long-term line transect and photo-identification data were analyzed to consider intra- and inter- annual patterns of habitat use and the underlying drivers for foraging areas in Clayoquot Sound, British Columbia. Time series, autocorrelation and weighted means analysis were used on the 20-years of data (1997–2016). A generalized additive model showed that whale use of the area was most strongly influenced by the maximum number of whales, and the date of its occurrence, recorded the previous year. This maximum, when it occurred in the summer, impacted the whale numbers for up to four subsequent years. The annual average number of whales per transect also influenced the proportion of whales known to return in multiple years to forage. Many of these returning whales first used the site to wean and returned in subsequent years to feed. The transect data was also used to contextualize passive acoustic recordings, comparing call type and rate for periods when the whale number, location and social context was known. Calling patterns appeared to be socially derived, with shorter-range knock calls dominant when whales were closer, and lower-frequency moans when foraging occurred when whales were more distant from each other. This suggests that prey-finding and site use may also be influenced by conspecifics.

## 1. Introduction

Analysis of baleen whale habitat use highlights areas that hold critical life history roles, where resources determine survival or reproductive success, or locations where social-sexual interactions are undertaken. Site importance is underscored by repeated use. Individual loyalty to an area may also indicate partitions in a population (e.g., [1,2,3]). However, changing ocean regimes or prey availability can alter long-standing habitat use patterns (e.g., [4,5,6]). 

Here we consider gray whales (*Escrichtius robustus*) foraging on the west coast of Vancouver Island to explore the significance of both top-down (predator-related) and bottom-up (productivity and ocean condition) forcing, and the influence of habitat variables on foraging. Prey declines have been observed in gray whale primary foraging areas in the Bering and Chukchi Seas. These also extend to their more southerly secondary foraging areas [7,8,9,10,11]. Observations suggest that whales are increasing their use of alternate foraging areas, and entertaining a more varied diet [10,12,13,14]. Reduced benthic prey biomass in the Chirikov Basin was thought to underlie the 1999–2000 Unusual Mortality Event, defined as a significant die-off of members of a marine mammal population (UME; [12,15,16]). Similar factors may be contributing to more recent observations of emaciated whales and UMEs (2019–2022; [17]). Although ecosystem change [18] and climate variability [11] are also likely contributing to altered habitat use. 

Gray whale foraging in Clayoquot Sound, on the west coast of Vancouver Island, has been noted since the mid-1970s [19]. Initially, foraging in this area was focused on benthic amphipods [20]. A shift in the principal food source and foraging areas in the mid-1990s was followed by a decline in this resource [21,22,23]. Currently, the primary prey species are epibenthic mysid swarms that inhabit shallow waters (~10 m water depth or less) on rocky reefs and in kelp [22,23,24,25]. Previous work in Clayoquot Sound has shown the nature of the predator-prey relationship, and the influence of annual removal on prey reserves [25]. Work has also shown foraging whale movements on a site-wide [26] and regional scale [24] to exploit dense prey swarms. Finer scale analysis has determined prey density thresholds that elicit foraging behaviors in gray whales [24,27]. Detailed examination of prey samples has also help define life history and repopulation potential of the primary prey species [28].

Gray whale foraging in Clayoquot Sound is dominated by members of a population sub-group that utilise a network of tertiary feeding sites along the migration route, known as the Pacific Coastal Feeding Group (PCFG; [29,30]). For designation to this subgroup whales must use sites between northern California and southeastern Alaska in more than one year for foraging from June 1 onward, which is typically documented through photo-identification (IUCN, 2018). Individual whales have returned to these feeding locations repeatedly, with some returning annually [29]. Despite this site fidelity, questions remain about the drivers of inter- and intra-seasonal foraging site use. 

Dedicated line transect surveys over a twenty year period are used to quantify foraging whale presence, with photographic mark-recapture data used to measure individual whales’ site fidelity. We examine the annual return rate of individual whales and site residency time, and its relationship to the number of foraging whales sustained at the site. In addition, we apply a fine scale patch-to-patch analysis of the spatial arrangement, and within-season movements of whales exploiting prey aggregations. We scrutinize whale movements in the study area through the summer, and the separation between individual whales as they feed. As well, time-series analysis shows the patterns of use and drivers between years. 

Acoustic recordings of foraging whales also formed part of our analyses. For some baleen whale species, calling during foraging is limited (e.g., [31,32]), presumably to focus energy on prey capture. However, others use acoustic signals to coordinate foraging between conspecifics (e.g., [33,34,35,36,37,38]). Here, we question whether acoustics form part of the multi-scale, multi-modal decision processes in the selection of prey patches on meso- to fine-scales [39]. We consider calling patterns as they relate to location and social dynamics to look for indications of how acoustics may guide habitat use. 

Finally, we consider how differing productivity and ocean environmental variables influences the number of foraging whales, likely through impacts on the prey base. Lab-based experiments have shown that temperature and salinity, for example, had an effect on post marsupial growth and sexual maturation in mysids [40,41,42,43], the principal prey species in Clayoquot Sound. Variability in these measures was therefore correlated to whale presence. Furthermore, upwelling strength and timing, that might further influence mysid maturation, reproduction or swarming were analysed.

Considering the influence of predation pressure and environmental variables on annual gray whale use of Clayoquot Sound may advance our appreciation of the driving forces in habitat selection further from the initial conclusions presented by Burnham and Duffus [25], and advance our understanding of the mechanisms used by the whale population over their full range. It will add to knowledge of the drivers of gray whales’ site fidelity, and the factors that underlie the use of this area by the PCFG population. As alternate foraging site use by gray whales increases, an appreciation of factors influencing site selection may help to predict the sites that will continue to be used, and identify future, as yet unused, foraging sites.

## 2. Materials and Methods

**Figure 1:**
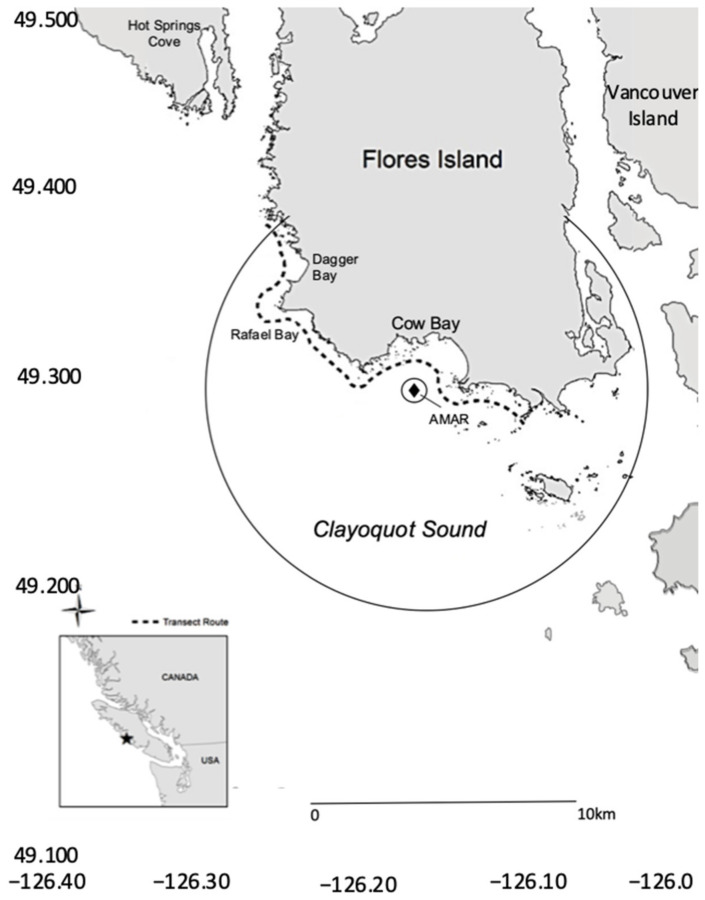
Study site map, showing transect survey route and Autonomous Multichannel Acoustic Recorder (AMAR) deployment location in Cow Bay (49.25629, −126.15928). The likely detection radii of gray whale class three moan calls are also shown. The AMAR location is the black diamond labeled ‘AMAR’ at the center of the detection circles. The smaller circle represents the range of detection exceeded 90% of the time (500 m) and the larger circle the maximum range 10% of the time (9 km).

Clayoquot Sound is a gray whale foraging site on the west coast of Vancouver Island, British Columbia (Figure 1). The study area is spatially discrete, bounded by deeper prey-free areas [22,23]. This analysis of gray whale feeding utilized three data collection techniques; regular systematic line transect surveys, mark-recapture photo-identification, and passive acoustic monitoring. 

### 2.1. Study Site Transect Surveys

Line transect surveys determined the number and location of foraging whales in the study area. Surveys were conducted approximately every 2–3 days, weather permitting, between 24 May and 6 September 1997 to 2016. A minimum of thirty transects were completed annually. The survey route was established after three years of intense observation, prey, and fecal sampling [22,23]. The 30 km survey route approximately followed the 10 m isobath along the southwest coast of Flores Island (Figure 1). It passed through principal prey habitat and provided visibility over the entire study area. The survey focused on rocky reefs and headlands that host mysid shrimp swarms, the principal prey. It also passed over amphipod beds, and areas where planktonic crab larvae aggregate [22,23]. The vessel travelled at a constant speed of 13 km/hr (~6 kts). Four dedicated observers maintained a 360 degree visual coverage around the vessel, in addition to a dedicated vessel operator. Surveys were aborted if visibility was below 500 m in any direction, or if sea state exceeded Beaufort 3 [22,23,25]. 

Whale behavior was determined from surface observations. Foraging was determined when a whale repeatedly surfaced and dove in the same location, focused on a prey patch. Surfacings were not more than 60 m apart, which is the average prey patch length [24]. Upon surfacing, a few inter-ventilations are used to recover from the previous dive and prepare for the next, which could be several minutes long. Upon locating a foraging whale, a time stamp and location were taken, using the position that the feeding dive began [25]. If foraging whales were observed off the transect line, the vessel approached them to obtain location data, and then returned to the transect where it had been paused. If a dive location was not possible, for example in unnavigable reefs, a range and bearing were taken to adjust the vessel position to accurately situate the whale. Whales were noted as either individuals or groups. A group of whales was identified as a number of individuals feeding in the same prey patch, within the average prey patch length [24], and/or displaying coordinated diving within a few body lengths of each other. In this case the same location was attributed to all members of the group. Cow-calf pairs were treated as a special case of the group category. They were identified as two whales with coordinated dives, and a marked size difference between the whales. Cow-calf pairs were counted as only one foraging whale. 

The main foraging site in the study area is Cow Bay (Figure 1). The locations of foraging whales in the bay recorded in the summers of 2015 and 2016 transect surveys were matched to the acoustic recordings made in those years. This established the social context of the calls heard concurrent to the visual observations. Straight-line nearest-neighbor distances between each location of foraging individuals or groups were derived to examine fine scale spatial dynamics. These formed an inter-individual, distance-based analysis for each transect. This formed a matrix of distance values between each individual or group of whales, and defined the social setting of each transect for comparison to the simultaneous acoustic records. It created a snapshot of the social context of foraging matched with acoustic data for approximately an hour (the transect transit time through Cow Bay). Again, cow-calf pairs were treated as a single entity, and single locations were used for grouped whales when they were foraging in the same prey patch, or when diving and surfacing locations of whales were undifferentiated from other whales.

### 2.2. Photo-Identification Mark-Recapture 

Identification photographs of a whale’s right and left side flanks were taken for all foraging whales for 1997–2000 and 2008–2013. No photographs were taken in 2001, and opportunistically taken on most surveys from 2002–2007 and 2014–2018 (see [44]). Individual pigmentation patterns and markings, distinctive knuckle profiles, and dorsal humps were used to identify individuals. This helped to determine the residency time and movements of each whale each summer, and its return rate between summers, as described by Calambokidis et al. [45]. Photographs were matched manually within the same season for residency calculations. Matching to a master catalogue compiled from 1997 established the rate of return. Return whales fulfil the definition set out by the IUCN [26] describing whales seen in more than one summer. Due to the lack of consistent effort through the years, the return rate and the number of returning whales represents a minimum value. Return whales are distinguished from whales that are only seen for one foraging season, referred to as single-visit whales. The number of years an individual returned since its first sighting was used to measure site fidelity. 

### 2.3. Inter- and Intra-Annual Gray Whale Habitat Use 

We examined the influence of top-down pressure and abiotic variables on the annual average whales per transect. Variables included the number and timing of the peaks in whale numbers measured by the maximum number of whales per survey per year, and the peak date, expressed as days elapsed from May 24 (Table 1). The mean number of whales per transect was derived for each year, as well as a global average for all years, which served to classify years as ‘high’ (above the global mean) or ‘low’ (below the global mean) whale years. Previous analysis by Burnham and Duffus [25] used measures of skew and kurtosis to describe the distribution of predation pressure level through the summer. Skew demonstrated the temporal distribution of whale abundance, or degree of symmetry of the number of whales around the peak date, to determine if the greatest predation pressure was earlier or later in the summer. We hypothesised that the greater the predation pressure in the latter part of the summer, the more impact it would have on the number of foraging whales sustained in the following year or years. Kurtosis described the shape of the peak compared to a normal distribution, to determine if the maximum number of whales recorded for that year was the true upper limit, or whether it was short lived, or not consistent with the transects preceding and following the peak day (Table 1). In addition, the proportion of returning whales and single-visit whales compared to the total number of individuals identified per year was calculated from the photo-identification surveys. Here we advance the analysis by Burnham and Duffus [25] statistically, and consider the potential influence of environmental and wider scaler oceanographic conditions.

Previous work has shown that average daily solar radiation and spring upwelling were not significant factors in the foraging use of Clayoquot Sound [46,47]. This analysis tested the first 12 years of the surveys, with no significant relationship established between the number of foraging whales and the upwelling index. There was also no relationship found between whale presence and the spring sunshine output between 1997 and 2006 [46,47]. We extended this analysis by considering the upwelling index values at latitude 48° and 51°, the date of the spring transition to an upwelling system (Spring Transition Index, STI), and time between this date and the transect (Table 1). Variables reflecting climate variation were assessed through the inclusion of the Pacific Decadal Oscillation (PDO) and sea surface temperature (SST) anomaly values. These variables are annual values calculated from monthly departures from the long-term means, and were included in this analysis to characterise the water properties. Oceanic conditions can influence gray whales [11] and their mysid prey [40,41,42,43]. These variables not only indicate temperature and salinity but also the stress imposed on the system by departures from the average (Table 1). An average SST value for the year was also included. 

Each variable was included with a lag of at least one year, following the conclusions of the initial predator-prey study [25]. The previous analysis indicated that intense foraging for a summer season necessitated at least one year of reduced predation for the prey population to recover, and so the perpetuation of this hypothesis was tested again here. This analysis builds on the work of Burnham and Duffus [25] by considering these regional scale variables that may influence bottom-up and environmentally driven processes, as well as the top-down influence that gray whales have on their mysid prey [25].

### 2.4. Statistical Analysis of Transect Data

The initial examination of the data included plotting each of the variables as a time series. The time series data for foraging whale number, both within- and between-years, was examined using autocorrelation and partial autocorrelation analysis. The analysis was performed in Python using the Pandas and matplotlib libraries in Jupyter notebooks (https://jupyter.org/, accessed on 1 August 2020). The correlation of each observation to previous observations was calculated with 95% confidence intervals. We hypothesise that foraging in the latter part of the season influences the prey-base in the following season, but foraging in the earlier part of the season could equally influence the number of whales that could be sustained later in the summer. To consider this we looked at the influence of factors with direct dependence. This considers whether previous observations have any direct bearing on the next, or if the number of whales seen on one transect could directly shape the number of foraging whales sustained in the study area. Indirect factors were considered using partial autocorrelation. Patterns or trends in the residual forecast errors resulting from a naïve or persistent forecasting model were examined. This would indicate that the model inputs did not account for all the variables influencing the output (number of foraging whales). The model was trained on 66% of the data and tested on 34% of the data. The distribution of residual errors was examined through density, histogram and quantile plots and summary statistics. The Dickey–Fuller test of stationarity was used to determine if repetitive temporal patterns were present. This would highlight patterns of whale use within and between seasons. The independence of whale observations between years was also tested by searching for autocorrelation between transect and environmental variables. These were tested with concurrent observations and with lags using the Box-Ljung statistic, performed in SPSS. Between-variable correlations were also considered with cross correlation analysis, with lags of up to 10 years tested following the time-series analysis. 

The influence of each input variable (Table 1) was initially tested through non-parametric Spearman’s Rank correlations between each of the variables. Then the independent relationship of each variable on the number of foraging whales that were observed in Clayoquot Sound was explored using a Generalised Additive Model (GAM). This was performed in Python using the linear GAM for the PyGAM library in Jupyter notebooks. This aids the interpretation of the influence of the variable without the influence of the other whale- or environment-based variables.

Spatial use of the study area through a summer season by foraging whales was visualized by mapping the locations of whale observations and deriving a heat map from the number of whales observed monthly at that location from May until September in QGIS 3.6 (Open Source Geospatial Foundation 2022). To track trends over time, weighted means of latitude and longitude values, weighted by whale number, were also calculated in Python and mapped in QGIS for each year. 

### 2.5. Passive Acoustic Recording and Analysis

An Autonomous Multichannel Acoustic Recorder (AMAR, JASCO G3A, Jasco Applied Sciences, Halifax, NS, Canada), with a calibrated GeoSpectrum M8E-132 omnidirectional hydrophone (sensitivity −165 dB re 1 V/µPa, effective bandwidth 5 Hz–150 kHz, gain of 6 dB; GeoSpectrum, Dartmouth, NS, Canada) was deployed in Cow Bay (49.25629 N, −126.15928 W), in approximately 20 m of water (Figure 1). This location was mid-bay, and balanced its proximity to feeding patches in both mysid and relict amphipod habitat, and distance from sources of wave and surf noise. Passive acoustic recordings were made continuously in frequencies up to 4000 Hz between 6 May and 14 September 2015 and 30 May to 5 September 2016.

A systematic aural and visual (AV) inspection of recordings was made for 20% of the recordings using Raven Pro Interactive Sound Analysis Software (Cornell Lab of Ornithology, Ithaca, NY, USA). Spectrograms were generated using a 256-point Hann window FFT with 50% overlap. A full 24-h period on every fifth day of the deployments, starting from the first full day, was analyzed for calls. In addition, recordings made at the time of transect surveys were also analyzed. For these periods, whale number, location relative to the recorder and each other were generated. Correlations between the rate of calling (number of calls per hour per whale present) and the number of whales foraging in the bay were considered. In addition, the total number of foraging whales recorded for each transect was also correlated with the call number for each call type, to examine any potential influence on calling from whales outside Cow Bay. To further refine our idea of social context, the calling data were compared to the average distance between individuals and groups calculated as part of an inter-individual, distance-based analysis. Considering average nearest-neighbor distance for whales inside Cow Bay and the presence of whales more widely in Clayoquot Sound observed on the full length of the transect helped to quantify the social aspects of calling for nearby whales in the bay, and on a broader site-wide scale. This examination was done for each call type, to test the hypothesis that call use changes based on social setting, while considering the number and proximity of whales. Temporal distribution of calls was also considered.

Calls were identified and categorized using call types described by Dahlheim [48] and attributed to one of the core call classes: class one knocks; class two sweeping tones; class three moans; or class four rumbles. Class one knocks are formed from a series of tones, and have been described as within-group, social calls [48,49,50]. This contrasts with lower-frequency class three moan calls that prevail in recordings during migration [51,52,53,54]. These are the most frequently reported call types, however the presence of class two up- or down-sweeping tones and rumbles with ‘zipper like’ qualities characterizing class four were also part of this analysis. The use of different call types may be indicative of the cooperative or competitive nature of foraging whales in close proximity, for example, or the spatial extent over which whales are communicating and perhaps sharing prey information. 

Ambient noise levels were derived for every minute of the recordings to estimate the detection range of whale calls from the recorder. Using the ambient noise levels (NL), and source levels (SL) from moan calls, taken from Guazzo et al. ([52]; 156.9 ± 11.4 dB re 1 µPa @ 1 m) in a repeated Monte Carlo simulation, estimating the likelihood a call of certain SL made at a certain distance would be heard at the recorder at a received level (RL) greater than the ambient noise level determined using the equation RL = SL – TL (r). Transmission loss of calls as they propagated was estimated by assuming spherical spreading loss, with little loss from attenuation due to the low frequency of the call. Although there are sound propagation variables unaccounted for, this detection range estimate was only used to establish whether calls likely originated within Cow Bay, and whether our comparison of visual surveys to the acoustic record was appropriate. 

## 3. Results

### 3.1. Foraging Whale Presence 

**Figure 2:**
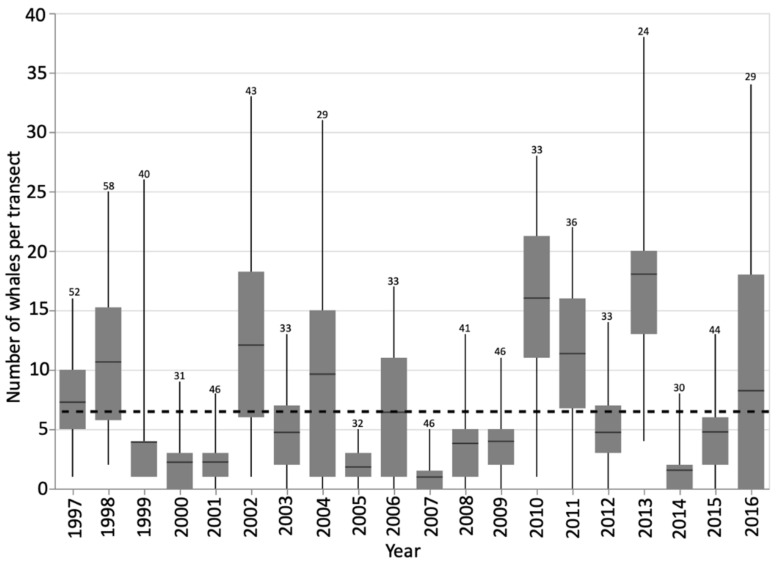
Boxplot to indicate the number of foraging whales sighted per transect survey in the study site. This is used as a proxy of foraging intensity through the analysis. The overall global mean of all years is indicated by the dashed line at 7.1 whales. The numbers above each box indicate the number of transects that year.

During the study period, 1997–2016, 759 transect surveys were completed and 1261 whale locations were taken for foraging individuals or groups. A global average from all transects in all years was calculated to be 7.1 whales/survey (Figure 2, Figure S1 in Supplementary Materials). This value was the threshold from which to make comparisons of the annual average number of whales per survey, and qualitatively describe years as either having a high (greater than global average) or low (lower than global average) number of foraging whales. Typically a ‘high’ year was followed by a ‘low’ year. If two consecutive years were classified as high (1997–1998 and 2010–2011) they were followed and/or preceded by at least one ‘low’ year (Figure 2). 

Photo-identification efforts catalogued 237 individuals, of which 122 were observed foraging in more than one summer. The remaining 48.52% were denoted as single-visit whales [44]. The number of years a whale was observed following its initial sighting averaged 4.5 years, and ranged from 2 years to 12 years for the study period [44]. 

**Figure 3:**
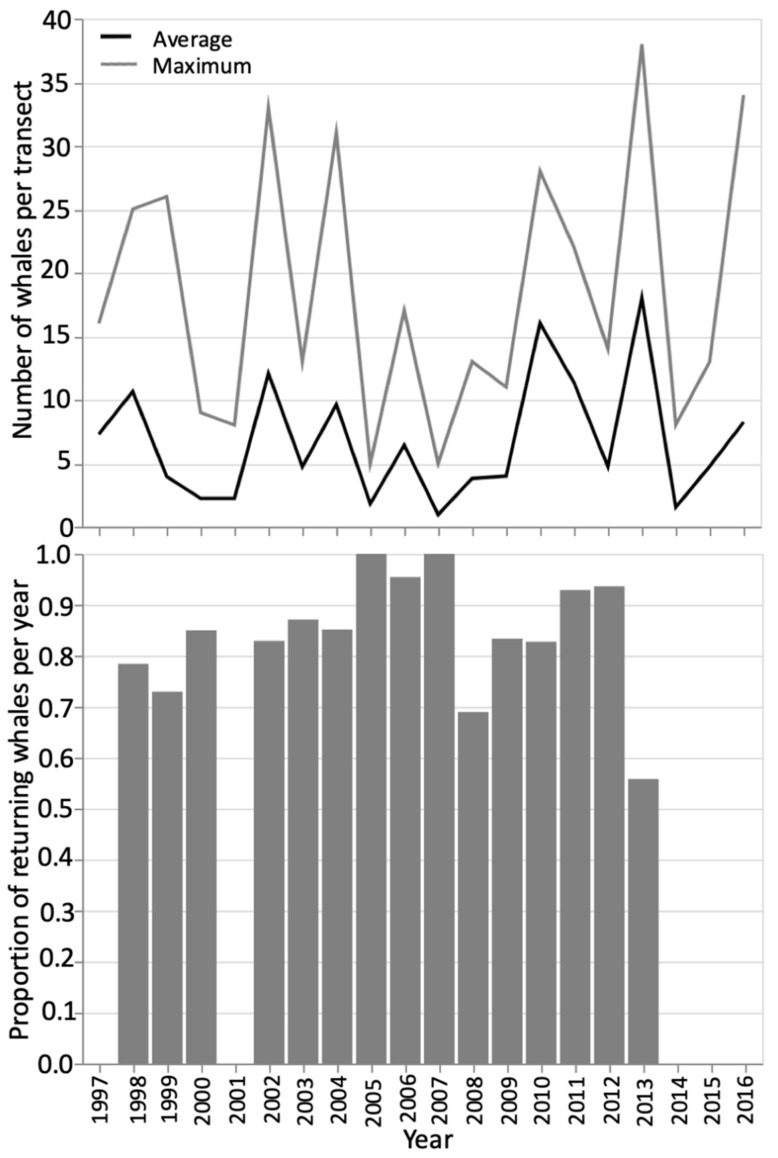
Above: The average number of foraging whales per transect over the summer season (‘Average’, black line) and maximum number of foraging whales in a single transect (‘Maximum’, grey line). Below: The proportion of the whales sighted in a year that were ‘Return’ whales and had been seen in previous years. No photo-identification data for this analysis for 1997, 2001, or 2014 onwards.

The non-parametric correlations of the number of whales per transect within a year showed that the number of whales on a transect was most strongly correlated to the number of whales in the previous transect (r_s_ = 0.795, *p* < 0.001), with transects typically conducted a few days apart. The number of whales seen on each transect was also significantly correlated with the annual average number of whales per transect (r_s_ = 0.599, *p* < 0.001) and the maximum number of whales (r_s_ = 0.506, *p* < 0.001) for that summer. These variables themselves were also strongly linked (r_s_ = 0.882, *p* < 0.001; Figure 3). As per the analysis by Burnham and Duffus [25], the distribution of whales through time, and their presence around this peak in whale number was examined through skew and kurtosis values. Foraging pressure was typically greatest in the early to mid-summer, before the peak date when whale numbers were at their maximum (right-handed skew, Figure S1 in Supplementary Materials). Peak dates were generally between mid-July and mid-August. In years with lower foraging whale numbers, whale presence tended to be focused more around this peak data, indicated by greater kurtosis. This contrasted with higher whale years, where kurtosis values indicated a platykurtic distribution often peaking in the later summer (Figure S1 in Supplementary Materials). This means that for ‘low’ years, foraging is more focused in time, with most whales present in the mid- to late-summer when the prey swarms have stabilized. 

**Figure 4:**
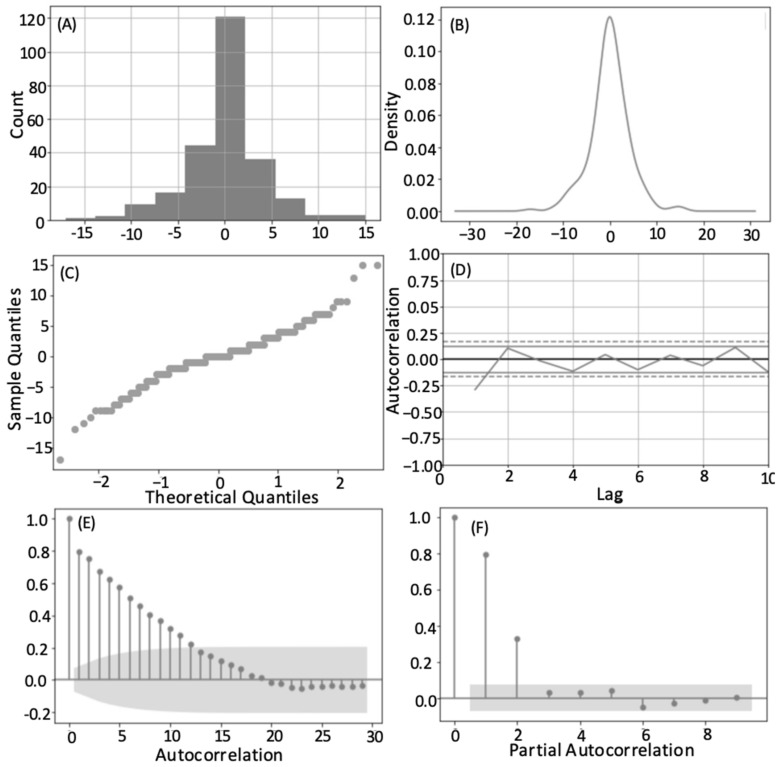
Histogram (**A**), density plot (**B**), Q-Q plot (**C**), lag analysis (**D**), autocorrelation and (**E**) and partial autocorrelation of residuals (**F**) for all transects in all years.

The time series analysis of all transect data showed a normal Gaussian distribution, confirmed by the normal sigmoidal curve of the Q-Q plot, with a slight left handed skew around zero (Figure 4). Calculating the residuals showed that the observed and predicted values differed little per transect and had a negative bias. Stationarity was determined by using a Dickey–Fuller test (test statistic = −6.100226, *p*-value < 0.000001, critical test statistic values 1%: −3.439, 5%: −2.866, 10%: −2.569). Therefore, within a season, the time of the observations from the line transect itself did not influence the number of whales per transect and no temporal trends or seasonality was detected. As well as testing the relationship between transects within a season, this aggregation of data (all transect all years) tests, as Burnham and Duffus [25] did, the influence of the number of whales present at the end of a season to the number of foraging whales seen in the following spring. This considers the winter and spring part of the study period where some prey species are able to reproduce and recover their swarm density and abundance [27], and considers the data continuously. This may have contributed to the variation in the forecasts. In the intra-annual analysis we considered the finer resolution of factors that hold foraging whales in the study area within a summer. The shift from positive to negative autocorrelation was similarly placed for each of these years at approximately eight. Many of these years were high whale years, and most showed a notable decline after the peak number of whales for that season to few or no whales per transect (Figure S1 in Supplementary Materials). This change occurred approximately 8–10 transects from the end of the season, and is approximately the peak date of the annual forging whale number. The partial autocorrelation also showed that any transect to another, non-consecutive, and with a lag, likely had other variables unaccounted for by the model acting on it. Non-consecutive transects were therefore much less similar than those that followed each other in time. 

All years’ residual values centered around zero, except 2015, which centered around −1. Considering the results for each year, generally a normal distribution was seen, however transect results from 1997, 2002, 2013 were multi-modal. These years are categorized as ‘high’ whale years, and most had an annual average whale number notably above the global average (Figure 2). In high whale years, the peak in foraging was not as strong, as seen with the kurtosis value, and greater numbers of foraging whales are sustained later into the summer. Almost every other year from 2002 onwards (2002, 2004, 2006, 2008, 2009, 2011, 2012, 2013, 2014) showed a similar autocorrelation pattern to that seen for the results when aggregating all transects in all years (Table 2, Figure 4E). This may be the overall year-on-year effect, captured by our coarse classification of ‘high’ or ‘low’ year whale year. 

**Figure 5:**
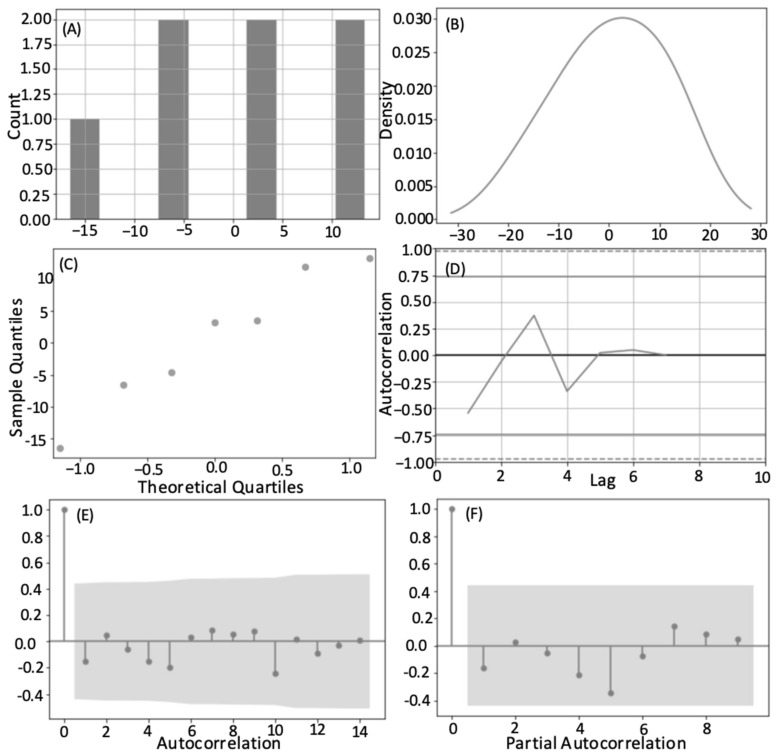
Histogram (**A**), density plot (**B**), Q-Q plot (**C**), lag analysis (**D**), autocorrelation and (**E**) and partial autocorrelation of residuals (**F**) for annual average number of whales per transect.

The time series analysis was also conducted for all transects by year to look for inter-annual patterns. This speaks more to the repetitive annual use of this area. The annual mean number of whales per transect in a yearly time series was used to represent each year. This also showed a normal distribution in the density and Q-Q plots, but a multi-modal distribution in the histogram (Figure 5). This highlights the oscillation in the data suggesting that a year of high predator removal necessitates a year of lesser foraging pressure to restore prey swarms [25,27]. The full time series of transect data showed that a year of high whale presence was typically followed by at least one year of reduced predation (Figure 2). The non-parametric Runs Test showed a non-significant result for both the annual average number of whales per survey (*p* = 0.164) and the proportion of returning whales (*p* = 0.110), again suggesting a ‘high-low’ patterning between years (Figure 2 and Figure 3). The test of autocorrelation indicated that the years with the most highly correlated average number of foraging whales were 3 or 4 years apart. This suggests that the repopulation by mysid prey may take longer than first suggested by the analysis of Burnham and Duffus [25]. A Related Samples Wilcoxon Signed Rank Test showed the annual average number of foraging whales was significantly correlated at the 0.05 level to the annual maximum number of whales, the number of individual whales identified, and the annual values of skew and kurtosis that describe the distribution of whale numbers through time. The strongest contemporaneously cross-correlated values were the annual average number of whales per survey and the maximum number of whales seen on a transect (0.874 no lag; Figure 3), and the date that this occurred (−0.500 no lag). This suggests that years with a higher average number of whales had a greater maximum number of whales and an earlier peak, but that a higher level of foraging could likely continue throughout the summer. This test again confirmed that lags of four years were typically the next strongest coefficient between variables. Strong correlative values also confirmed the relationship with data lagged by one year (average number of whales per transect lagged 1-year, 0.730, and maximum number of whales per year, lag-1 year 0.627). 

**Figure 6:**
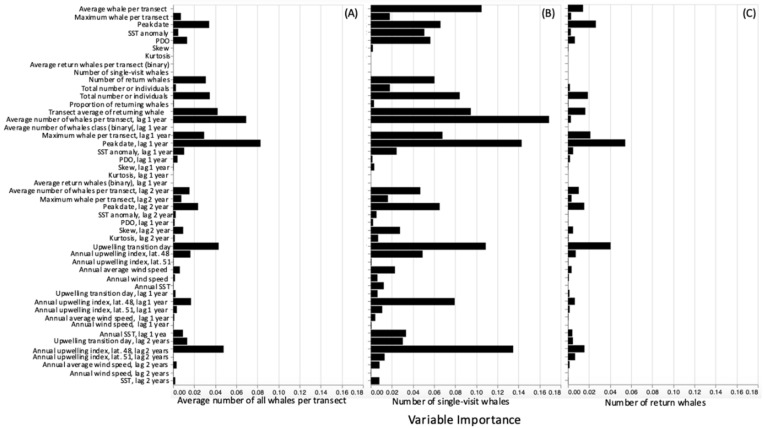
Relative importance of whale-derived and environmental variables in determining the annual average number of whales per transect (**A**), the number of returning whales (**B**) and the number of single visit whales (**C**) derived from the cross-correlation analysis.

The GAM helped clarify the importance of whale- or environment-based factors on the number of whales using the site annually, using the annual mean value. The site use of single-visit and returning whales was also considered separately. Generally the peak date in the year prior was the most influential on the number of foraging whales sustained in any year. This confirms our hypothesis that the later the maximum number of foraging whales is seen, the more impact it will have in the following year due to top-down forces (Figure 6A). This outweighed the importance of the average or maximum number of whales. These were seen to be more influential to single visit whale numbers (Figure 6B). In-year whale variables were also ranked higher for single visit whales, all of which suggests that an opportunistic foraging strategy was employed by these whales. Environmental conditions at a broader spatial scale, highlighted by the influence of both inputs from latitude 48° and 51°, and a longer time scale were found for single visit whales (Figure 6B). For returning whales, the importance of variables was much less, suggesting that there may be a factor we have not captured that promotes site fidelity. Concurrent and previous years’ environmental conditions could influence the growth rate and maturation of mysids following removal by whales, with their relative variable’s importance suggesting that it is the prey’s ability to recolonize that is mediating between the top-down drivers suggested previously [25], and the bottom-up pressure that is thought to dominate marine systems. In all cases the transition date of the year being considered was the most influential environmental variable (Figure 6). This may be due to the initiation of growth and swarm stabilization each year.

### 3.2. Residency and Return Rates

Photo-identification indicated that some whales displayed multi-year site fidelity to Clayoquot Sound. Whales returning to forage comprised a high proportion of the individuals identified during the years of dedicated mark-recapture effort, with some of the highest proportions found in low whale years (Figure 3, Table 3). An increased proportion of single-visit whales was found during high whale years ([44], Figure 3, Table 3). The number of single-visit and returning whales were both significantly positively correlated with the number of individual whales seen each year (r_s_ = 0.681, *p* < 0.001 and r_s_ = 0.970, *p* < 0.001, respectively, Table 3), but the total number of whales and proportion of re-sighted whales was significantly negatively correlated (r_s_ = −0.531, *p* = 0.042, Figure 3). The greater the annual average number of whales per transect, the greater the number of individual whales documented through the summer (r_s_ = 0.850, *p* = 0.004; [44]). Lower proportions of re-visiting whales in earlier years were more likely a representation of the establishment of a capture-remark catalogue rather than whale site use. 

There was a strong significant positive correlation between mean annual residency time of an individual, and the annual average number of whales per transect (r_s_ = 0.933, *p* < 0.001). This again highlights the difference between the high and low whale years, and that this difference is not just in the mean and maximum values of the year, but how long foraging whales were sustained in the season. The mean residency time of whales was 23 days per year (range, 1–113). There was an average difference in residency time of 10 days between single-visit and returning whales ([44], Unpublished Data). Mother’s with calves typically spent the longest time in the study area, and had an average residency of 44 days. A significant positive correlation was also found between a whale’s residency time and its return rate; those whales sighted in multiple years were most likely to forage for extended periods (r_s_ = 0.419, *p* < 0.001; [44]). The likelihood of a calf returning to Clayoquot Sound in the years subsequent to weaning increased as residency time in that first year increased (r_s_ = 0.669, *p* < 0.001; [44], Unpublished Whale Lab Data).

### 3.3. Spatial Use by Whales 

**Figure 7:**
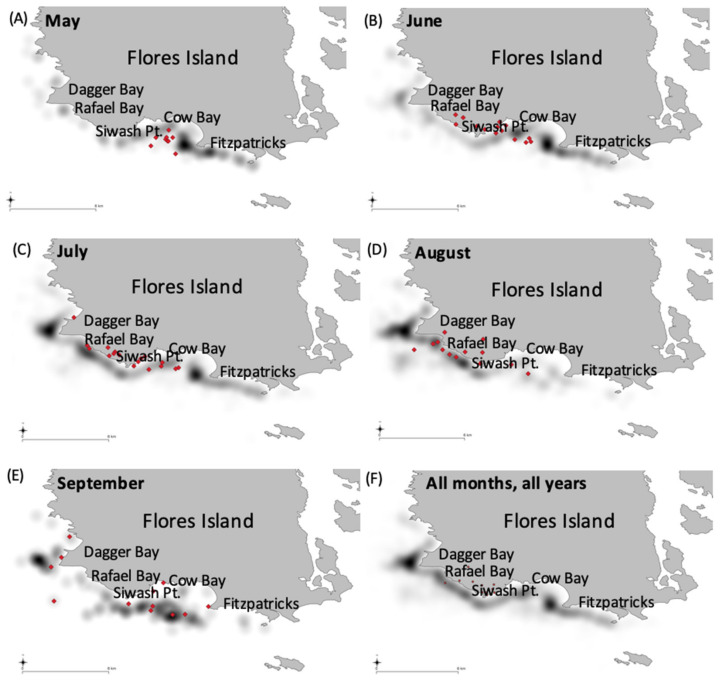
Heat map of foraging whale locations from the transect data by month (Black shading). Weighted average mean locations are shown for each year (red diamonds). Data from all transect and all years in (**A**) May, (**B**) June, (**C**) July, (**D**) August, (**E**) September, and an aggregate of all years, all months (**F**) are also shown.

The location data from the transect surveys indicated that whales did not move through the study area randomly to exploit prey patches, but moved from south to north as the summer progressed. The monthly weighted means and heat map of whale locations showed Cow Bay to be the main feeding site in Clayoquot Sound in May (Figure 7), although foraging whales were present in all months. The weighted mean locations moved increasingly north each month, with August showing foraging focused around Rafael and Dagger Bays (Figure 7). Prey sampling has showed that these areas served as ancillary prey aggregations from late July to Mid-August, where crab-larvae are opportunistically exploited (Authors’ Pers. Obs.; [22,23,26]).

Finer-scale foraging patterns in Cow Bay were considered from thirty-four transect surveys in 2015 and thirty in 2016. A total of 135 individual or whale group locations were used to evaluate the distances between individuals and groups as they foraged. Seven transects in 2015 found no whales in the bay, and two transects in 2015 and four transects in 2016 showed a single whale in the bay with no inter-individual distance calculated. The matrix of distance observations showed greater distances between whales when the number of foraging individuals was increased. However, the number of groups also increased, as did the distance between groups, as group numbers increased (Figure S2 in Supplementary Materials). The average number of whales per survey was 4.8 at a mean distance of 544 m between individuals or groups for the transects in 2015 and 5.8 whales at a mean distance of 130 m in 2016. The number of cow-calf pairs in Cow Bay was higher in 2015 than 2016. While they generally use areas away from the main foraging corpus [54], the distance between cow-calf pairs and other foraging whales increased significantly as the number of whales increased (2015, individuals r_s_ = 0.773 *p* = 0.002, groups r_s_ = 0.832, *p* < 0.001; 2016, individuals r_s_ = 0.881 *p* < 0.001, groups r_s_ = 0.871, *p* < 0.001, pairs r_s_ = 0.754, *p* = 0.003).

### 3.4. Passive Acoustic Analysis 

A total of 2073 h of recordings made during the summers of 2015 and 2016 were manually analyzed. A total of 5751 calls (2015: 2795, 2016: 2956) were heard. Call rates and distributions did not differ significantly between the years (Mann–Whitney U at the *p* = 0.05 level, Figure S2 in Supplementary Materials), and so the data from the summers were aggregated. Calls matched the core types described by Dahlheim [48] and ‘motherese’ calls described almost exclusively in the breeding lagoons [53,54,55,56,57,58]. Motherese calls comprised 20.5% of all calls, but have only been recorded between mothers and calves, and so were not included as part of the analyses of acoustic behavior of foraging whales (instead see [54]). For the remaining 4574 calls, class one knock calls were the most frequently recorded, accounting for 31.5% (n = 1089) of all the identified calls. Class three moans were the next most commonly heard call type (28.2% of all calls, n = 1622), followed by sweeping tones of class two (24.7%, n = 1130). Very few class four rumbles were recorded, comprising less than one percent of all calls (0.627%, n = 13) (Figures S2 and S3 in Supplementary Materials). 

**Figure 8:**
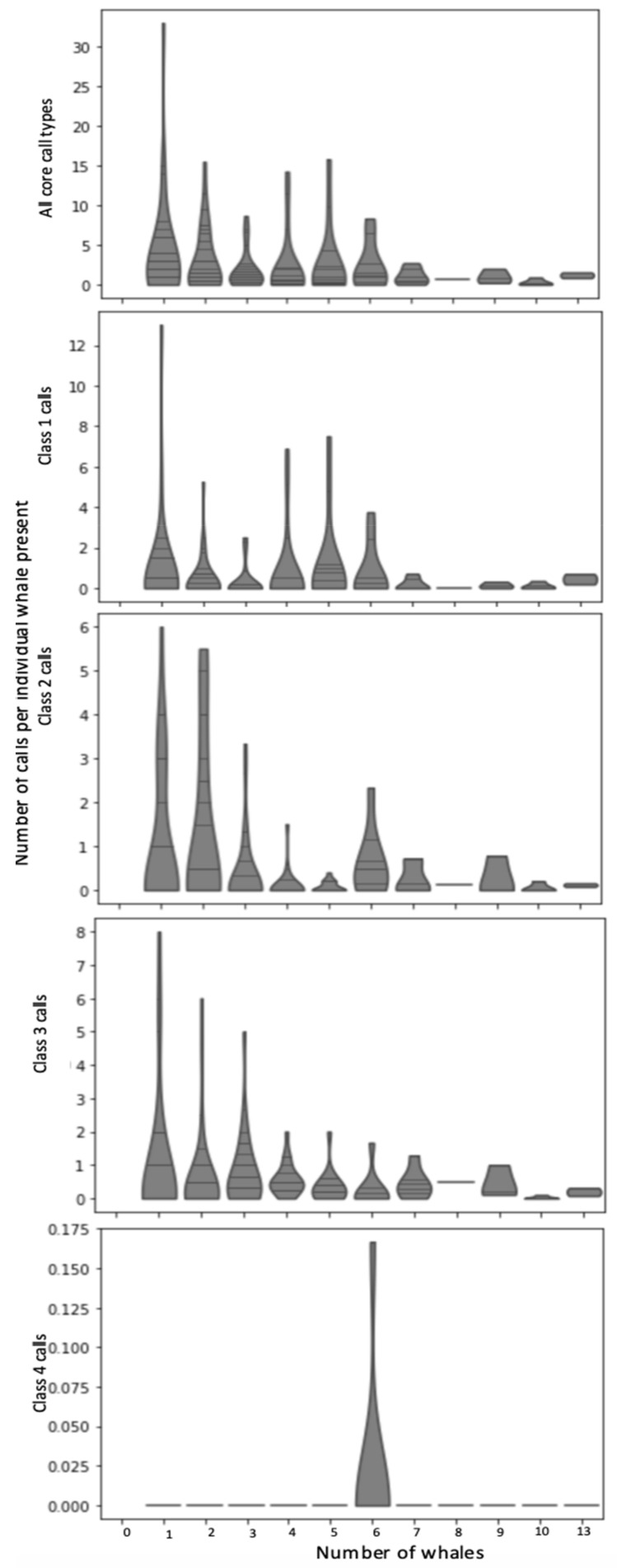
Calling rate (calls per hour per individual) for core call types compared to the known number of whales in Cow Bay for that hour, taken from 243 h of observation data. Call class one knock calls, class two sweeping tones, class three moans, and class four rumbles are shown aggregated and separately.

Comparing the manual verifications to the transect data showed the number of calls/hour/individual for each call type was greater when fewer whales were in the bay. Indeed, calling was highest when only one or two foraging whales were present (Figure 8, Figure S4 in Supplementary Materials). The calling rate (calls/hour/individual present) did not vary significantly between call types, with similar means and standard deviations (class one, $\bar{x}$ = 0.634, s.d = 1.662; class two, $\bar{x}$ = 0.651, s.d. = 1.339; class three, $\bar{x}$ = 0.639, s.d. = 1.232). However, class one call use showed greater variance (class one = 2.764, class two = 1.793, class three = 1.518) in the calling rate. Class four rumble calls were excluded from further analysis due to the paucity of calls (class four, $\bar{x}$ = 0.001, s.d. = 0.014, variance = 0.0002). Class one knocks have been described as a social call. The use of this higher-frequency, shorter-range call type was greater when four or five foraging whales were present (Figure 8). Class one knocks were more frequent in 2016 in recordings during transect surveys. For 2016, the whale number was higher and the average distance between individuals/groups was smaller. Their rate of use, and call number/individual was high when the distance between individuals and groups was lower (Figures S4 and S5 in Supplementary Materials). 

The converse pattern was true for class three calls. The hourly rate of class three moans/individual declined as the number of individuals increased (Figure 8), and it was most frequently heard when the distance between individuals and groups was greater (Figure S5 in Supplementary Materials). The sweeping tones of class two increased in use when six whales were present for feeding (Figure 8), and was employed when whales had both short, and greater inter-individual or group distances (Figure S4 in Supplementary Materials). In this analysis the use of up- and down-sweeps were not distinguished. 

The number of class two and three calls/individual/hour was also significantly correlated with the number of foraging whales on a full transect (class two, r_s_ = 0.361, *p* = 0.001; class three, r_s_ = 0.348, *p* = 0.002). This was examined to test whether whales might be communicating between bays. The hourly call rate of core call types was not significantly correlated with the number of cow-calf pairs present (r_s_ = 0.095, *p* = 0.255), further supporting their separation from the foraging whales and use of motherese.

For all types, calling significantly declined as the summer progressed, however this was concomitant to foraging whales abandoning Cow Bay as a feeding location. The estimated detection range from ambient noise suggested that although the maximum extent of calls (10% of the time) could reach 9 km, the recordings were limited to an approximate 2 km range around the AMAR, which allows coverage of the full extent of the bay. At times with the highest noise levels, detection range could be limited to approximately 500 m, however, the recordings analyzed exceeded this range 90% of the time (Figure 1). This confirmed that comparison between the visual and acoustic data, albeit it rudimentary, was appropriate. 

## 4. Discussion

The re-analysis of transect survey data from Clayoquot Sound showed that the number of foraging whales per transect was most influenced by the number of whales present in the previous days, and strongly correlated to the average number of whales per transect and the maximum number of whales observed in that year. On an inter-annual scale, the conclusions made by Burnham and Duffus [25] were corroborated, however this analysis highlighted the need for conditions that strengthen prey repopulation and recovery. The impact of previous years’ foraging was still highlighted, with the peak date in the previous year ranking highly as an influential variable. 

Prey sampling in Clayoquot Sound during the timeframe of this study, but not detailed here, has shown that peaks in foraging whale number in the late summer can reduce the overwintering mysid population, which influences the foundation from which the swarms can repopulate the following spring. Only one of the thirteen species present in Clayoquot Sound consistently reproduces over winter [28]. The timing of the foraging pressure and extent of this pressure per year, here quantified by the maximum number of whales, the number of different individuals, the peak date, and the annual values of skew and kurtosis, could in fact be influential for up to four subsequent years, as demonstrated by the autocorrelation analysis. This is a longer time scale than used in the GAM or considered in previous works [25]. A four year lag time may be needed for full repopulation of prey swarms. Two sets of three consecutive years of lower whale foraging effort were found in the full time series (1999–2001, 2007–2009, Figure 2). Therefore, we suggest that annual prey removal by gray whales propels several years of reduced foraging, which then creates a substantial recovery in swarm size and density (2005–2011, Figure 2). The mechanism of inter-annual mysid repopulation has been described by Burnham [28]. Their ability to reproduce and re-establish swarms likely mediates annual foraging effort. 

The environmental variables, upwelling strength and timing (STI), and the variation in sea surface temperature and climate (PDO), primarily influenced the year being considered (Figure 6). System productivity is typically greatest if upwelling is initiated earlier in the year. Years with the spring transition (STI) in mid-March to early April showed the highest annual number of foraging whales, with peaks between mid-July and mid-August (Figure S1 in Supplementary Materials). A later STI could delay the first spring mysid brood and the subsequent swarm growth [28]. The survival, growth, and age at sexual maturation for mysid species is temperature and salinity dependent. Together these variables have been described as ‘ecological abiotic master factors’ [43]. Mysid species have demonstrated limited tolerance to altered conditions [40,41,42,43]. 

Years that sustain a higher number of foraging whales typically have a greater proportion of single-visit whales (Figure 3) that are opportunistic users of Clayoquot Sound [44]. The increased importance of whale number in previous years, especially of returning whales, to the number of whales seen annually, illustrates the site-loyalty shown by gray whales over the 20-year time period. This is also indicated by the high proportion of returning whales across the time series despite the varying number of individuals and average number of whales per transect (Table 3, Figure 2). Maternal-recruitment, culture, and memory could influence the number of whales returning to forage year-on-year. Cow-calf pair presence was consistent across all years. The photo-identification also showed several calves that were weaned in this area have subsequently returned in following years to forage, with at least one example seen of the calf bringing its own offspring to wean in Clayoquot Sound ([44,54], Unpublished Whale Lab data). 

Site use and residency patterns in Clayoquot Sound (48.52% of catalogued whales known to return for multiple years) are similar to those reported for the full PCFG range by Calambokidis et al. ([29]; 51.9% resighted more than once). The movement between areas may demonstrate gray whales’ sensitivity to influences on a range of scales, perhaps being able to assess relative prey availability on regional and local scales [24,39]. As Clayoquot Sound is one area in a complex of feeding sites [29], the relative number of foraging whales between sites may indicate where prey reserves are greatest. The single-visit whales, which may be whales that forage in other areas of the PCFG or in the primary Arctic feeding grounds, perhaps foraged in Clayoquot Sound to take advantage of higher prey reserves, or to reduce the energetic burden of travelling further. It is in the low whale years, when prey abundance is reduced, and the number of individual whales is limited, that the site loyalty of PCFG whales to Clayoquot Sound is demonstrated (Table 3). A preliminary comparison of the Clayoquot Sound whales to a compilation of observations spanning southern California to Alaska showed that whales that repeatedly forage in the study site were most frequently seen in areas from southern Vancouver Island to northern Puget Sound when not in Clayoquot Sound ([29]; Cascadia Research Unpublished Data).

A threshold of mysid-density that draws and holds foraging whales has been described on a prey-patch, and site level [24,26,27], therefore the number of foraging whales can be considered a representative metric of mysid prey abundance. Within-season movements of whales foraging in Clayoquot Sound showed movements northwards as the season progressed (Figure 7). Weighted mean averages and heat maps of locational data and prey sampling not detailed as part of this study (see [47,59]) showed this annual movement pattern was driven principally by swarm abundance and maturation. The use of foraging locations around Rafael and Dagger Bays in July and August also likely resulted from whales taking advantage of crab-larvae aggregations. These larvae are a lipid rich, high calorie, but short-lived prey [22,23,60].

The inter-individual distance analysis in Cow Bay showed that as the number of whales increased, the distance between whales increased. This positive correlation held up to a maximum of six whales. The number of group associations and whales feeding in the same prey patch also increased (Figures S2 and S3 in Supplementary Materials). This also suggests, based on the mysid threshold for foraging whales established by Feyrer and Duffus [24], that the observation of a greater number of whales is likely driven by greater patch density. 

Acoustics informs foraging whales on a micro- to meso-scales [39]. Here, we hypothesized that calling may have a function in foraging. In other baleen whale species, calling during foraging aids cooperative foraging [34,59,61], locating and capturing prey [62], and in coordinated feeding techniques [61,62,63]. The estimation of detection radii confirmed that the comparison of visual and acoustic data was carried out on an appropriate scale, and that the observations from transect surveys could provide some information on the social context of calling. The differences in calling rate compared to social context suggests that this may be one of many factors in calling behavior. The use of call type differed with the number of whales (Figure 8) and the distance between individuals or groups (Figure S4 in Supplementary Materials), and strengthened the distinction in the use of class one calls, and class three moans when whales were more widely dispersed.

At their simplest, our findings add to what is known about gray whale calling while foraging. The dominant use of class one knocks agrees with what has been reported from Arctic foraging areas [10]. However, here we do not see the exclusive use of this call type as previous studies have reported [10,48]. We also see the use of class two sweeping tones and class three moans. The greater rate of class three moans in 2015, a low whale year, compared to 2016, a high whale year, suggests that these calls may be used to maintain contact between distant whales. Our location data also shows an increased average distance between individuals and groups as numbers increased (Figures S2 and S3 in Supplementary Materials). Class three moans are lower in frequency than other calls in the repertoire. They are employed during travelling and migration as a far-ranging call, and have been implicated in maintenance of contact with remote conspecifics during this time [51,52,53,64]. This further-propagating call type was also significantly correlated with the number of whales observed on the full transect and not just within Cow Bay, which suggests that whales could share information beyond the extent of the bay. Conversely, the class one knock calls have been described as within-group communications, used over shorter range, with possible social or sexual applications [48,50]. They were dominant when more whales had shorter mean inter-individual distances (Figure S3 in Supplementary Materials). This suggests that acoustic information may be shared on a local scale. 

We could speculate that the acoustic exchanges using these calls, which was increased in 2016 relative to 2015, was to share prey information on a reduced spatial scale by employing a higher frequency, modulated call. Call type is consistent with spatial density and distribution and here provides an initial view into the role that acoustics plays in foraging behavior. The use of class one knock calls perhaps shows an interaction of whales in the immediate vicinity of each other, within or between neighboring patches. However, in years or foraging sites where prey is depleted, the acquisition of energy may require wider searches. The use of longer-range class three moans may be one of several mechanisms used by gray whales to facilitate that. 

The relationship between calling rate (calls/hour/whale) and the number of whales was non-linear, despite the estimated detection radius largely being limited to the extent of the bay (Figure 1 and Figure 8, Figure S3 in Supplementary Materials). Previous visual-acoustic comparisons in calving lagoons by Ponce et al. [65] suggest that call frequency was linked to the number of connections within the network of communicating whales, rather than simply the number of whales (see Figures S3 and S4 in Supplementary Materials). Call number, then, may represent the number of whales an individual is actively exchanging acoustic information with. 

Calling rate was highest when whale numbers were lower, however the use of social class one knocking calls increased to a maximum when five whales were in the bay (Figure 5). This could signify that, in addition to the number of whales in proximity to each other, call type and rate may depend on other factors including the spacing between individuals and use of prey patches. The use of ‘social’ call types when whales were in closer proximity, the increased number of groups when whale numbers increased, and the complete lack of observations of competitive behavior over the length of our study (Authors’ Pers. Obs.) suggests that foraging is cooperative, and that calling during foraging may provide some of the information used to exploit prey. This fairly crude inter-individual distance comparison to the calls, however, likely does not capture the full complexity of the potential acoustic network or the function of calling in foraging. 

Gray whales are highly mobile and can move between foraging sites to satisfy their needs. They arrange themselves between our study site and adjacent foraging areas to take advantage of high resource levels and remain in areas that have sufficient prey [24,29]. Studies such as this one, over longer time periods and in spatially discrete areas, provide a useful insight into the role of predators in marine ecosystems, and the potential for predation to exert top-down effects between years. Large scale distribution patterns of marine predators are driven by food availability; at regional or local scales current and past predation shapes the prey field such that it determines the distribution of upper-trophic-level species [66]. This phenomenon of top-down control by a predator on its own prey source has been described for seabirds [67], marine mammals, pinnipeds [68] and intertidal communities [69]. As ocean regime change and mortality events become more frequent, and prey reserves are reduced, understanding the variables defining habitat use helps us understand the stressors to a population like the gray whales studied here.

## 5. Conclusions

The analysis of the PAM recordings hints that at least part of the whales’ ability to locate and exploit prey is derived from their acoustic sense. Yet, we acknowledge that this first foray into calling behavior and insights and speculation about call function leaves many unanswered questions about how the acoustic sense is used and what can be derived from call structure, type, and rate as it pertains to gray whale foraging. Furthermore, if the acoustic modality contributes to prey location and capture, the noise from vessels in much of the gray whale’s range may impact foraging and has yet to be fully considered [70,71,72].

A more complete understanding of gray whale use of Clayoquot Sound will be developed by considering more of the push and pull factors from adjacent regions in the wider PCFG range, including prey availability, stressors, and metrics that characterize oceanic regimes. A focus on returning PCFG whales will better define this subgroup and its critical foraging areas. Prey density and patch distribution are driving forces in whale presence and behavior, but it is possible that acoustic behavior, whether it is directed communication or passive listening, adds to the inputs used for site selection and residency time. 

In recent years, the eastern north Pacific gray whales have recorded two Unusual Mortality Events (UMEs) and it has been suggested that the population may be reaching carrying capacity [12,73]. Long time series data, such as that presented in this study, and predictive habitat modelling using long-term ecological data, will increase our understanding of gray whale foraging and add to our knowledge of how whales adjust to a changing preyscape and ocean conditions in broader terms. 

## Figures and Tables

**Table 1:** Variables included in the analysis.

Variable	Source
Skew	Calculated from transect survey data
Kurtosis	Calculated from transect survey data
Average return proportion	Transect survey and photo-identification data
PDO anomaly	Available at: psl.noaa.gov/pdo/ (accessed on 12 September 2022).
SST anomaly	Measures taken from Amphitrite Light station, available at: pac.dfo-mpo.gc.ca/science/oceans/data-donnees/lighthouses-phares/index-eng.html (accessed on 12 September 2022).
Sea surface temperature	La Perouse wave buoy (46,206): weather.gc.ca (accessed on 12 September 2022)
Wind speed	La Perouse wave buoy (46,206): weather.gc.ca (accessed on 12 September 2022)
Upwelling	48° and 51° latitude: coastwatch.pfeg.noaa.gov (accessed on 12 September 2022)
Spring Transition Index (STI)	48° and 51° latitude: coastwatch.pfeg.noaa.gov (accessed on 12 September 2022)
Average foraging whale number	Transect survey data
Peak foraging whale number	Transect survey data
Peak foraging whale date	Number of days elapsed from 24 May, transect data

**Table 2:** Results of the autocorrelation residual analysis for all transects in all years (All), for the annual average number of foraging whales per transect (Annual) and for all transects per year from 1997 to 2016.

Year	Count	Mean	Standard Dev.	Minimum Value	Maximum Value
All	248	−0.0161	4.155	−17.000	15.000
Annual	7	0.609	10.652	−16.508	13.314
1997	19	0.211	4.826	−12.000	9.000
1998	21	−0.571	8.500	−17.000	11.000
1999	14	0.5000	11.992	−25.000	24.000
2000	11	0.000	1.483	−4.000	2.000
2001	19	−0.105	2.923	−5.000	7.000
2002	14	−0.786	3.068	−5.000	5.000
2003	12	−0.250	3.494	−9.000	4.000
2004	11	0.364	2.014	−3.000	5.000
2005	11	0.091	1.221	−2.000	2.000
2006	12	−0.083	2.021	−3.000	4.000
2007	16	0.063	1.389	−3.000	3.000
2008	14	−0.500	2.79	−9.000	2.000
2009	16	−0.563	3.596	−9.000	3.000
2010	11	−0.455	8.054	−17.000	13.000
2011	13	−0.923	4.092	−7.000	7.000
2012	11	0.000	3.464	−6.000	4.000
2013	9	−0.889	8.594	−12.000	15.000
2014	11	−0.091	1.514	−3.000	3.000
2015	12	−0.250	1.288	−2.000	2.000
2016	10	0.000	0.943	−2.000	2.000

**Table 3:** The total number of individual gray whales identified per year by photo-identification, and the proportion of whales that returned for more than one year to forage.

Year	Total Individuals	Return Whales Proportion (%)	Classification
1998	51	78.4	high
1999	37	73.0	low
2000	40	85.0	low
2002	41	82.9	high
2003	62	87.1	low
2004	27	85.2	high
2005	6	100.0	low
2006	22	95.4	low
2007	7	100.0	low
2008	29	69.0	low
2009	48	83.3	low
2010	58	83.8	high
2011	56	92.9	high
2012	47	93.6	low
2013	95	55.8	high

## Data Availability

Data is part of ongoing student works. It may be made available on request from the corresponding author.

## Supplementary Materials

The following supporting information can be downloaded at: https://www.mdpi.com/article/10.3390/ani12202735/s1, Figure S1: Number of foraging whales per transect shown for every year in the time series with a line of best fit. Figure S2: The number of calls of each call type heard throughout the summer 2015 and 2106 recordings. Figure S3: Spectrograms to show examples of the four core call types: class 1 knocks, class 2 sweeping tones, class 3 moans, and class 4 rumbles. Figure S4: Comparisons of the number of individual and groups of whales, the number of links between individuals and/or groups, straight-line distance between individuals or groups and number of calls heard determined from the inter-individual distance and acoustic data in Cow Bay for 2015 and 2016 together. Individual observations and line of best fit are shown. Figure S5: Comparisons of number of individual and groups of whales, average distance between all individuals or groups, and the total number and call number per individual for class one knocks, class two sweeps and class three moans.
