# Supplementary figures and images for: A Multi-Dimensional Examination of Foraging Habitat Use by Gray Whales Using Long Time-Series and Acoustics Data
Source: Animals (Basel). 2022 Oct 12;12(20):2735. doi: 10.3390/ani12202735 (PMC9597834; doi:10.3390/ani12202735)

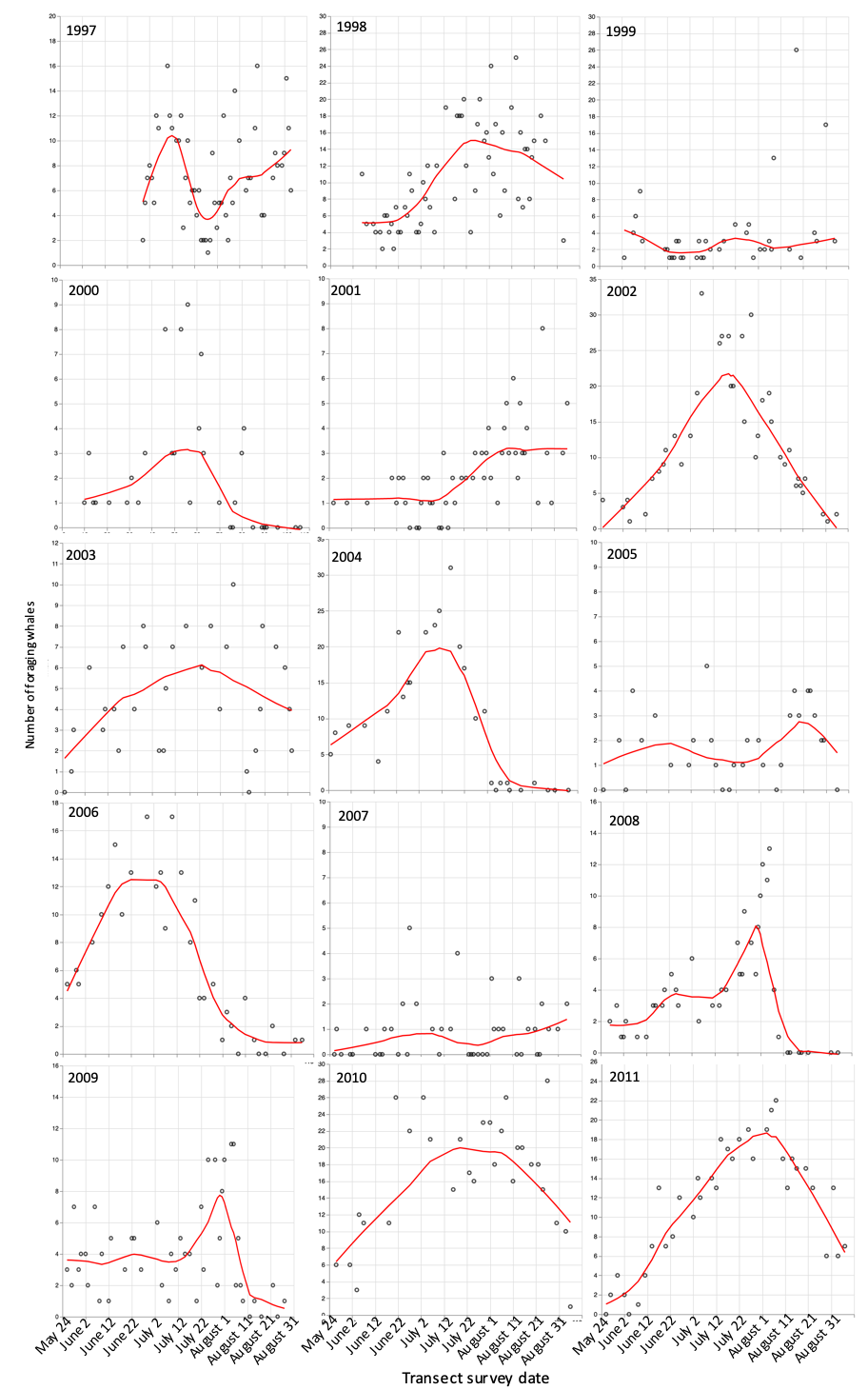

Supplement: Supplementary file 1 [file animals-12-02735-s001.zip › animals Figure S1a_redo.tiff]

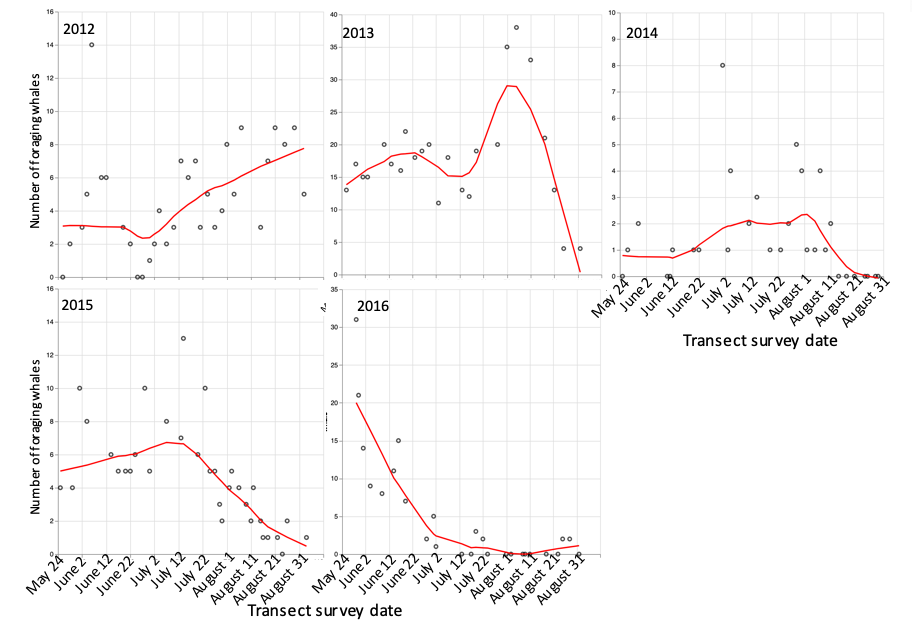

Supplement: Supplementary file 1 [file animals-12-02735-s001.zip › animals Figure S1b_redo.tiff]

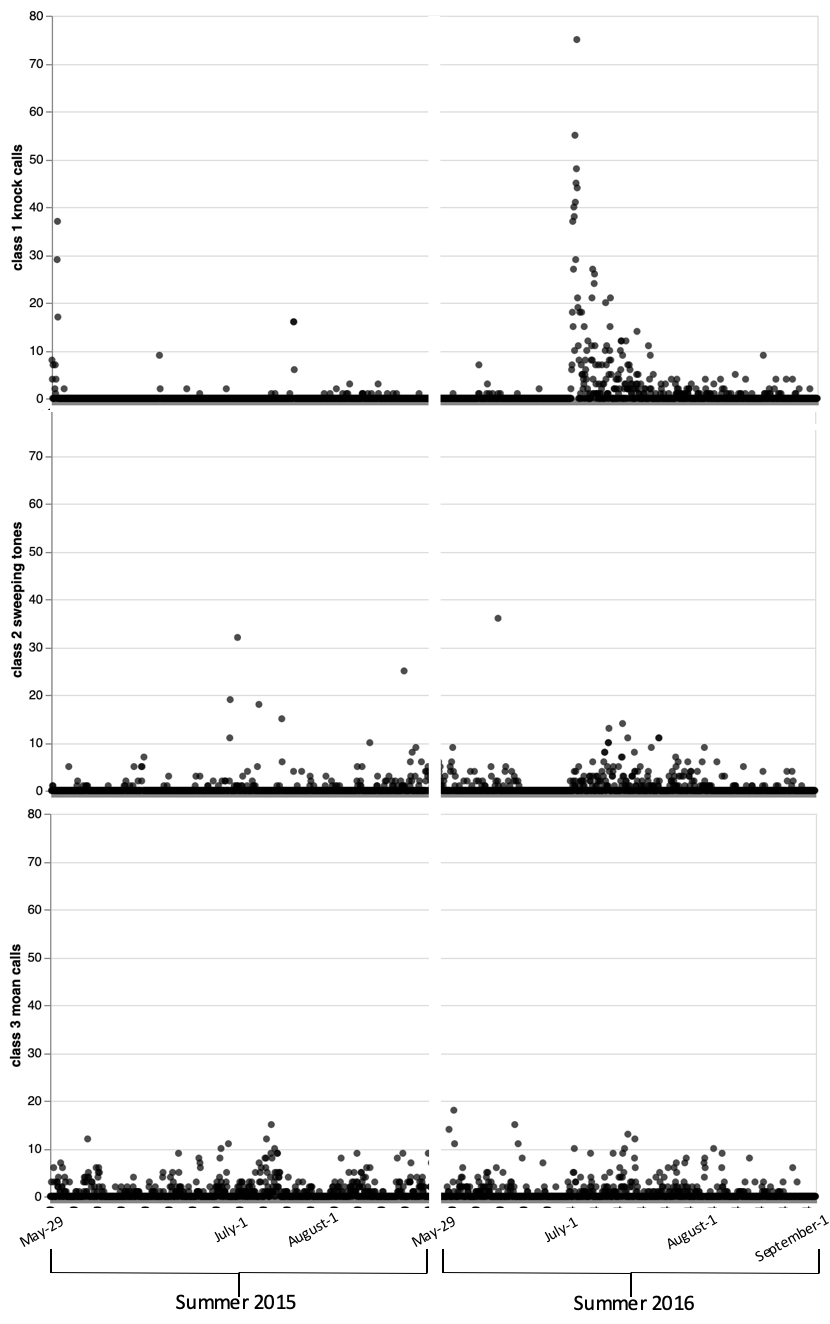

Supplement: Supplementary file 1 [file animals-12-02735-s001.zip › animals Figure S2_calls.tiff]

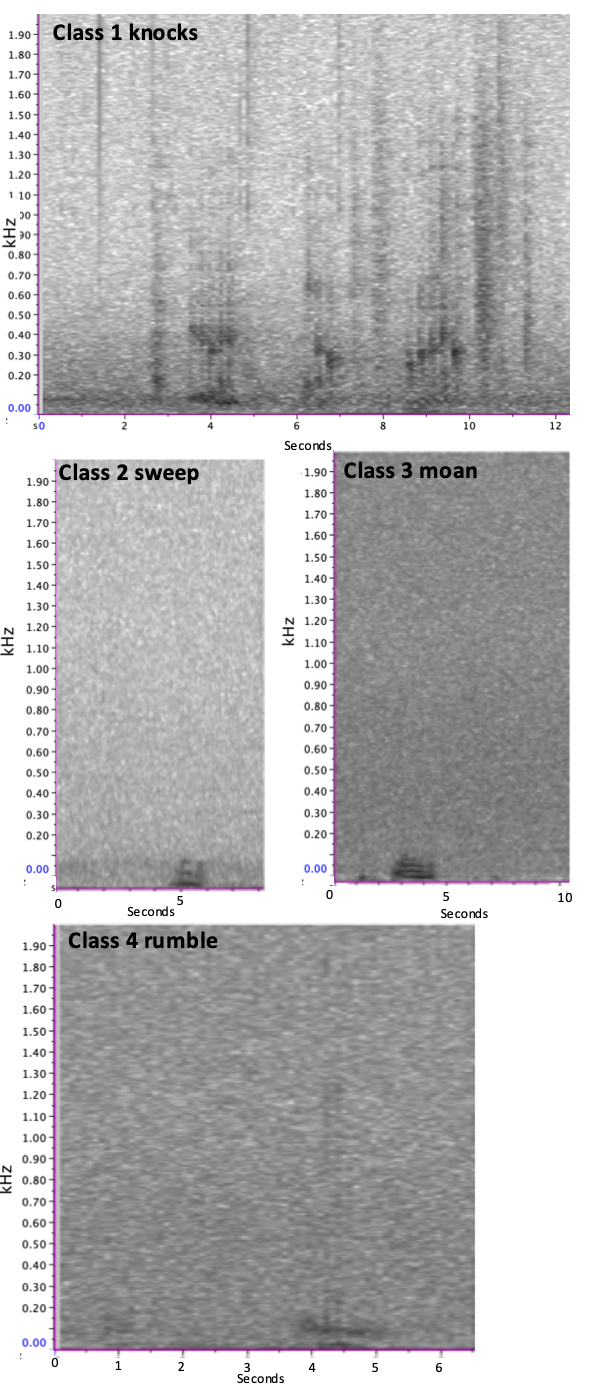

Supplement: Supplementary file 1 [file animals-12-02735-s001.zip › animals Figure S3_redo.tiff]

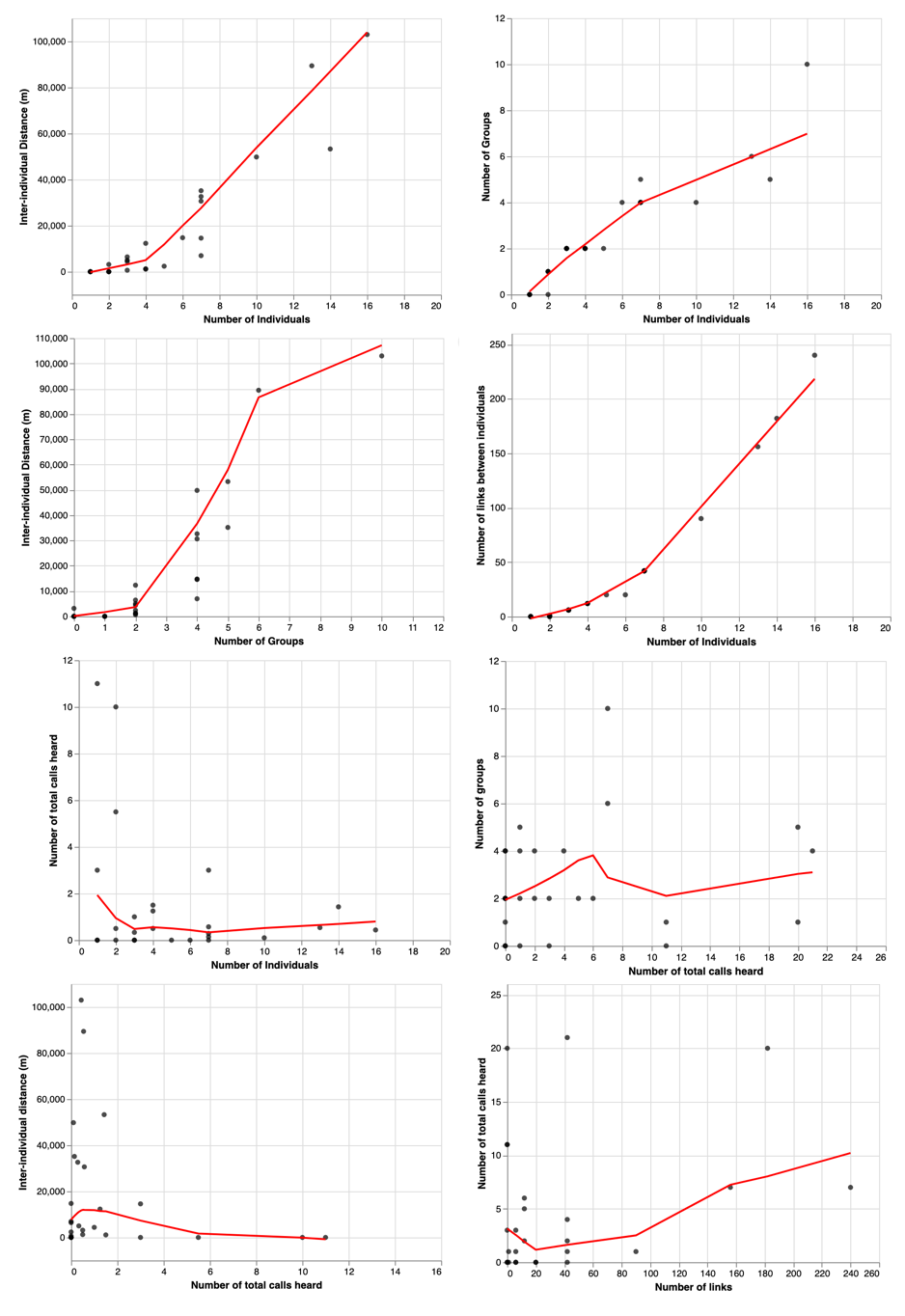

Supplement: Supplementary file 1 [file animals-12-02735-s001.zip › animals Figure S4_redo.tiff]

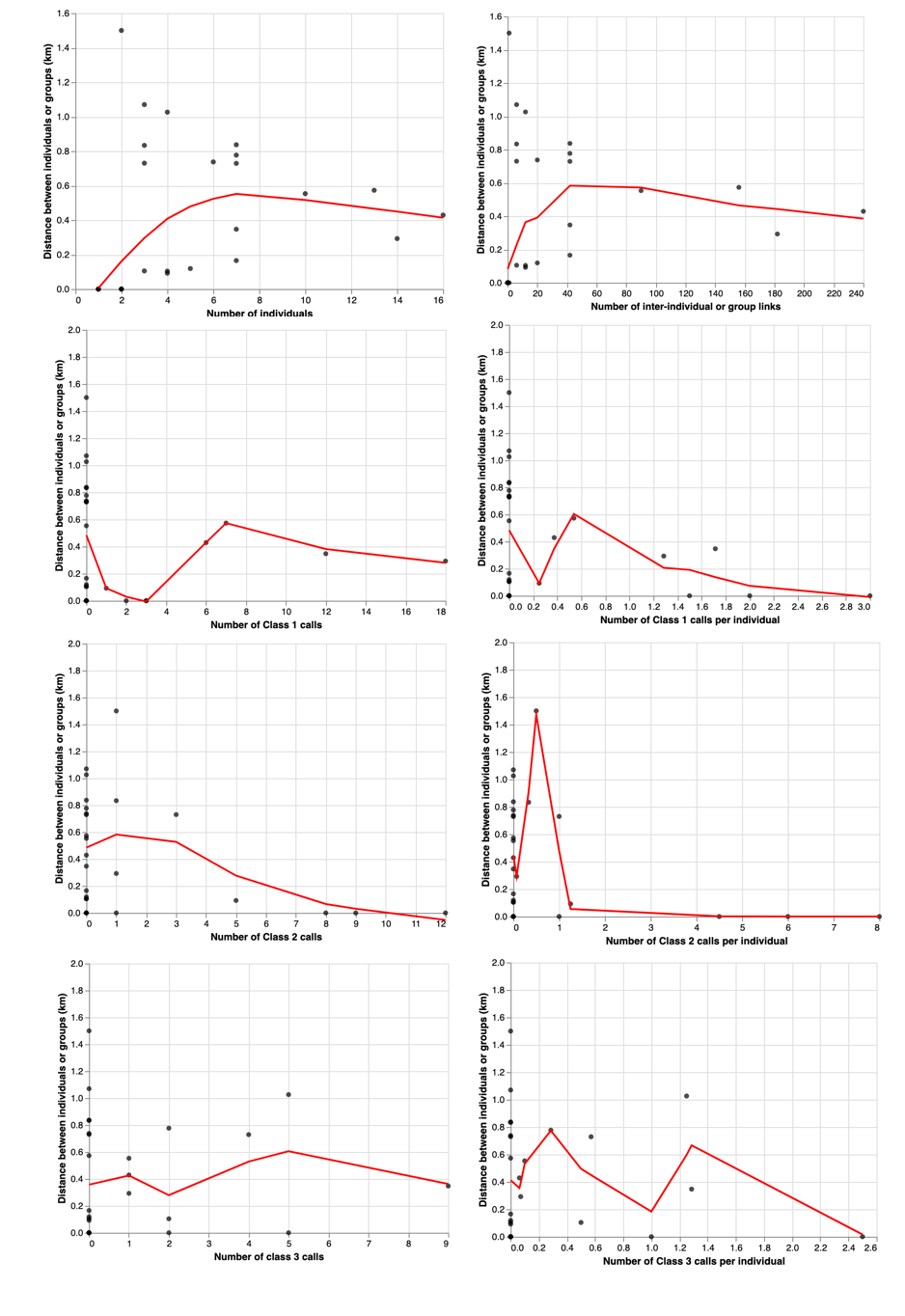

Supplement: Supplementary file 1 [file animals-12-02735-s001.zip › animals Figure S5_redo.tiff]
